# Silicon nanowires decorated with platinum nanoparticles were applied for photothermal-enhanced sonodynamic therapy

**DOI:** 10.7150/thno.58755

**Published:** 2021-09-03

**Authors:** Lina Sun, Xianwen Wang, Fei Gong, Kui Yin, Wenxiang Zhu, Nailin Yang, Shang Bai, Fan Liao, Mingwang Shao, Liang Cheng

**Affiliations:** Institute of Functional Nano & Soft Materials Laboratory (FUNSOM), Collaborative Innovation Center of Suzhou Nano Science and Technology, Soochow University, Suzhou, Jiangsu 215123, China.

**Keywords:** Silicon nanowires, Si-Pt nanocomposites, sonodynamic therapy, chemodynamic therapy, combined cancer therapy

## Abstract

Sonodynamic therapy (SDT) triggered by ultrasound (US) can overcome pivotal limitations of photo-therapy owing to its high depth-penetration and low phototoxicity. However, there is still a need to develop more efficient sonosensitizes to enhance the therapy efficiency.

**Methods:** In this study, Pt nanoparticles (Pt NPs) are reduced on silicon nanowires (SiNWs) by *in situ* reduction to prepare Si-Pt nanocomposites (Si-Pt NCs).

**Results:** Si-Pt NCs can produce reactive oxygen radicals (ROS) under ultrasound (US) irradiation, which have sonodynamic therapy (SDT) effect. Meanwhile, Si-Pt NCs can convert excess hydrogen peroxide (H_2_O_2_) into ROS in the tumor microenvironment, which endow strong chemodynamic therapy (CDT) effect. Taking the advantages of the mesoporous structure of SiNWs, the SDT and CDT effects of Si-Pt NCs are stronger than those of the pure Pt NPs and SiNWs. Besides, the mild photothermal effect of Si-Pt NCs further improves the SDT&CDT activity and realizes the combined cancer therapy.

**Conclusion:** The developed Si-Pt NCs with the ability of photothermal enhanced SDT/CDT combined therapy play a momentous role in the novel cancer treatment.

## Introduction

Although more and more scientists are devoting to cancer research, cancer is still one of the most serious causes threatening human health 1, 2. The traditional cancer treatments like surgery, chemotherapy, and radiotherapy, are the most common clinical strategies in fighting cancer. However, they also meet lots of issues 3-5. Sonodynamic therapy (SDT) has recently aroused widespread interests due to its noninvasion and much deeper tissue penetration, which is suitable for large and deep-site tumors 6-8. SDT can trigger sonosensitizer to generate toxic reactive oxygen species (ROS) *via* ultrasound (US) to kill cancer cells 9-14. At present, although the exact mechanism of SDT is not well determined, there are mainly three widely recognized mechanisms: US produces heat 15, ROS 16, or mechanical damage 17 that cause tumor cell death. Sonosensitizers play a momentous role in cancer treatment. Up to now, various kinds of sonosensitizers have been applied to realize SDT. For example, organic molecules such as hematoporphyrin, chlorophyll, and protoporphyrin IX derived from photosensitizers often cause low SDT efficacy due to the low chemical/biological stability 18, 19. Inorganic sonosensitizers like Ti-based nanoparticles have unique energy-band structure and relatively high chemical/physiological stability for SDT 20-23. However, traditional titanium dioxide (TiO_2_) nanoparticles have poor sonodynamic performance due to the rapid recombination of electrons (e^-^) and holes (h^+^) in the band structure. There are many strategies to improve the efficiency of ROS generation by promoting the effective separation of e^-^ and h^+^ including forming oxygen defect layers, combining TiO_2_ nanoparticles with other noble metals (such as Pt, Au, Ag) and doping metal irons (Fe, V) into TiO_2_ nanoparticles to overcome carrier recombination 24-28. There is still a need to develop more efficient sonosensitizes to enhance the therapy efficiency.

Owing to the rapid proliferation of cancer cells, changes in metabolic pathways and malformation of tumor blood vessels, the solid tumors often have a series of microenvironment characteristics which are different from normal tissues such as hypoxia 29, slight acid 30, 31, and high hydrogen peroxide (H_2_O_2_) 32. Tumor microenvironment (TME) not only promotes the development and metastasis of tumors, but also seriously affects the effectiveness of cancer therapy. Chemodynamic therapy (CDT) is defined as treatment using the Fenton reaction or Fenton-like reaction to generate hydroxyl radical (•OH) in the tumor site 33. For example, iron-based nanomaterials activate the Fenton reaction to produce •OH from H_2_O_2_ in TME, and then the generated •OH can result in cell apoptosis and inhibit the tumor growth. However, the Fenton or Fenton-like reactions are suppressed due to the low efficiency of CDT agents and complex TME 34, 35. Recently, platinum nanoparticles (Pt NPs) have been extensively reported as nanozymes with good catalytic activity and favorable biocompatibility 36, 37. However, their catalytic efficiency still needs to be further improved.

**Figure 1:**
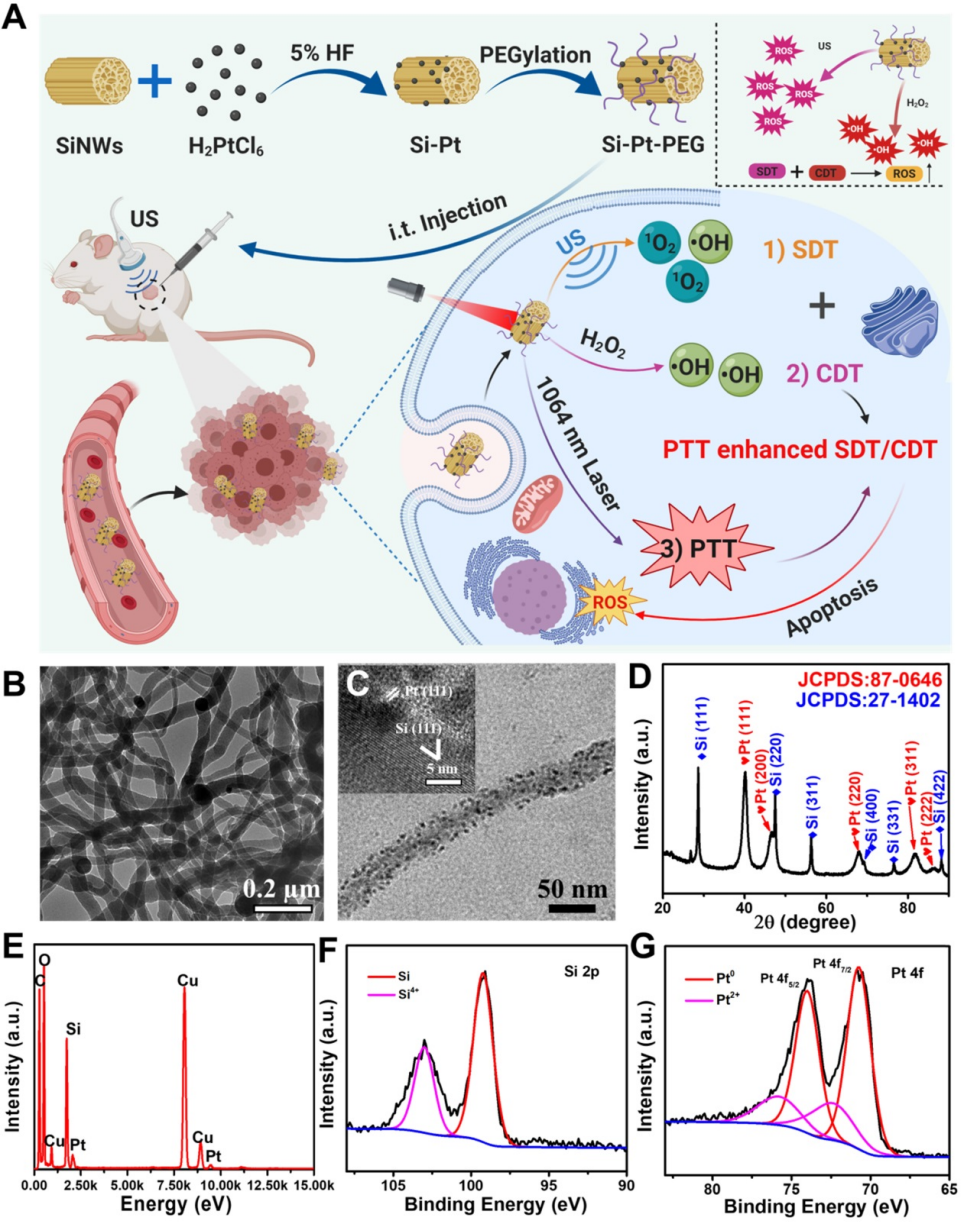
**Characterization of the as-prepared Si-Pt nanocomposites.** (**A**) Schematic illustration of Si-Pt nanocomposites (NCs) with unique photo and ultrasonic properties for photothermal enhanced sonodynamic therapy; **(B)** TEM image of SiNWs;** (C)** TEM image of Si-Pt NCs and HRTEM image of Si-Pt NCs (insert image); **(D)** PXRD of Si-Pt NCs; **(E)** EDX elemental analysis confirmed the coexistence of Pt and Si in the NCs; and **(F&G)** XPS spectra of Si 2p and Pt 4f peaks of Si-Pt NCs.

Silicon nanowires (SiNWs), as inorganic semiconducting materials, have been widely applied in electrocatalysts, nano-devices, and photovoltaics 38-40. The previous studies have shown that SiNWs have good biocompatibility and are capable of reducing metal nanoparticles on their surface *via in situ* reduction with no need for extra reductants, which endow the good properties of the Si-based nanostructures 41. Herein, we synthesized Si-Pt nanocomposites (Si-Pt NCs) by *in situ* reduction of Pt nanoparticles grown on SiNWs (**Figure 1A**). Due to the good catalytic activity of Pt NPs and mesoporous structure of SiNWs, the synthesized Si-Pt NCs had the good activity of SDT and CDT, much stronger than that of pure Pt NPs, which were capable for combine cancer therapy. In addition, the mild photothermal effect could significantly improve the combined SDT&CDT cancer treatment. Our work highlighted the great significance of the multifunctional Si-Pt NCs for application in the novel cancer therapy.

## Results and Discussion

SiNWs were prepared *via* a thermal-evaporation oxide assisted growth method 42, 43 and the Si-Pt NCs were constructed by *in situ* reduced approach 44. Briefly, ~5% hydrogen fluoride (HF) solution was added into SiNWs suspension and dispersed in pure water by ultrasonication. The Fourier transform infrared (FTIR) spectroscopy of SiNWs and SiNWs after HF treatment were collected **(Figure S1)**. The peak ranging from 2300 and 1900 cm^-1^ showed that the silicon-hydrogen (Si-H) bonds were formed on the surface of SiNWs. Pt NPs were* in situ* reduced on the surface of SiNWs using the reducibility of Si-H bonds. From the transmission electron microscopy (TEM) images, we found the synthesized SiNWs **(Figure 1B)**, and Pt NPs showed uniform morphology. The diameter of the Pt NPs was about 5-10 nm, which were attached to the surface of SiNWs **(Figure 1C, S2)**. After surface modification by PEG-SH, the hydrodynamic size of Si-Pt-PEG NCs was ~280 nm and showed excellent stability in physiological solutions even after 7 days **(Figure S3)**. High-resolution TEM (HRTEM) revealed that the lattice spacing was corresponded with Si (111) lattice plane and Pt (111) lattice plane, respectively **(insert in Figure 1C)**. Furthermore, it was shown that all the diffraction peaks of powder X-ray diffraction (PXRD) were in accordance with Si and Pt standard peaks (JCPDS cards, Pt, No. 87-0646; Si, No. 27-1402), indicating that the Si-Pt NCs were successfully synthesized **(Figure 1D)**. To further confirm the presence of SiNWs and Pt NPs, energy dispersive X-ray spectroscopy (EDX) displayed the distribution of Si and Pt elements **(Figure 1E)**, revealing that the molar ratio of Si and Pt was ~41:1, which was consistent with the results of inductively coupled plasma optical emission spectroscopy (ICP-OES). From X-ray photoelectron spectroscopy (XPS), the Si 2p peak was deconvoluted into two peaks at 103.3 eV and 99 eV, which were contributed to Si^4+^ 2p and Si 2p **(Figure 1F)**. In the Pt 4f spectrum, there were two kinds of Pt with the peaks of Pt^0^ at 70.7 eV (4f_7/2_), 74.0 eV (4f_5/2_) and Pt^2+^ at 72.4 eV (4f_7/2_), 75.7 eV (4f_5/2_), respectively** (Figure 1G)**, indicating that Si-Pt NCs were consisted of Si and Pt elements. All these results demonstrated that Pt NPs were reduced on the surface of SiNWs and formed Si-Pt NCs.

**Figure 2:**
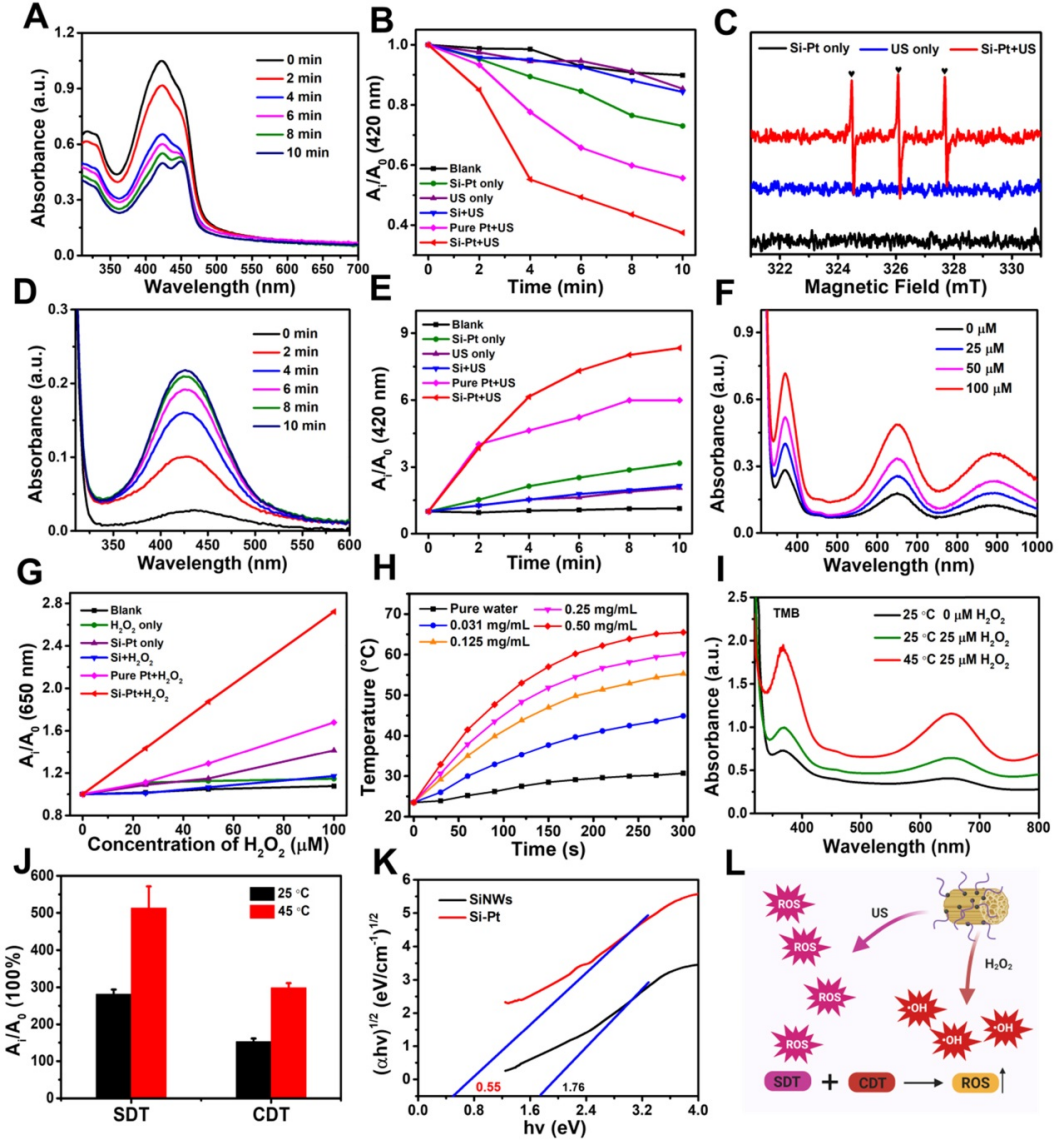
** Chemodynamic and sonodynamic performance of Si-Pt NCs**. **(A)** Time-dependent oxidation of DPBF indicating ^1^O_2_ generation by Si-Pt NCs under US irradiation; Comparison of **(B)** DPBF oxidation and **(E)** OPD oxidation by Si-Pt only, SiNWs only, and pure Pt under US irradiation for 10 min;** (C)** ESR spectra demonstrating ^1^O_2_ generation by Si-Pt NCs under US irradiation; Time-dependent oxidation of **(D)** OPD indicating •OH generation under US irradiation and **(F)** TMB indicating •OH generation with the addition of H_2_O_2_; **(G)** Comparison of TMB oxidation by Si-Pt NCs only, SiNWs only and pure Pt with the addition of H_2_O_2_; **(H)** Photothermal heating curves of Si-Pt NCs under 1064 nm laser irradiation (1.0 W▪cm^-2^); **(I)** The oxidation of TMB by Si-Pt NCs with the addition of H_2_O_2_ at different temperature (25 °C; 45 °C); **(J)** Chemodynamic and sonodynamic effect at different temperature (25 °C; 45 °C) under the control of laser irradiation; **(K)** The bandgap of SiNWs and Si-Pt NCs; and **(L)** The schematic of sonodynamic and chemodynamic capability of Si-Pt NCs.

Since the semiconductor materials of Si-Pt NCs, the electron and hole were easily separated under an external field, which could generate ROS. We wondered whether Si-Pt NCs could be used as sonosensitizers under US conditions. A singlet oxygen probe called 1,3-diphenylisobenzofuran (DPBF) was applied to detect the ^1^O_2_ generation contributed to US. Under the action of US, the featured absorbance peak of DPBF at ~420 nm incubated with Si-Pt NCs decreased gradually. Compared with Si-Pt NCs only, US only, Si + US, and pure Pt + US samples, Si-Pt + US group displayed the highest US-triggered ^1^O_2_ production efficacy and the highest efficiency time of generating ^1^O_2_ was at 4 min **(Figure 2A-B)**. Moreover, the SDT efficiency of Si-Pt NCs was compared with that of the commercial titanium dioxide (TiO_2_) nanoparticles. It found that Si-Pt NCs exhibited much better SDT efficiency than the commercial TiO_2_ nanoparticles, probably due to the unique structure of Si-Pt NCs **(Figure S4)**. Among all groups, Si-Pt + US group had the highest intensities of the DPBF, suggesting that Si-Pt NCs could be excellent sono-sensitizers that generating ^1^O_2_. Electron spin resonance (ESR) was also demonstrated the generation of ^1^O_2_ by Si-Pt NCs under US irradiation **(Figure 2C)**. To capture the •OH generation produced by SDT, o-Phenylenediamine (OPD) was employed as another ROS probe. With the increasing US action time, the absorbance peak of oxidized OPD at ~420 nm distinctly increased after OPD incubated with Si-Pt NCs. In contrast with the other control groups (Si-Pt only, the US only, Si + US, and pure Pt + US), Si-Pt + US group also exhibited the highest •OH generation efficacy, which was consistent with the time of ^1^O_2_ generating **(Figure 2D-E)**. The intensities of the Si-Pt + US group significantly increased, demonstrating that the abundant •OH generation in the process of SDT. Moreover, ESR spectra were used to identify the types of ROS and hydroxyl radical was also produced by Si-Pt NCs under US irradiation **(Figure S5)**. In addition, the ROS-generation abilities of pure SiNWs under the same US irradiation were much weaker than that of Si-Pt NCs **(Figure 2B, 2E)**, indicated that the presence of SiNWs could make the more remarkable SDT effect compared to pure SiNWs.

Since Pt NPs had the good catalytic ability, the CDT activity of Si-Pt NCs was investigated. 3,3',5,5'-Tetramethylbenzidine (TMB) was •OH probe that could be oxidized and turn blue during the enzymatic degradation of hydrogen peroxide by horseradish peroxidase. As shown in **Figure 2F**, a significant increase of the peak at ~650 nm was observed when TMB was incubated with H_2_O_2_ and Si-Pt NCs. Moreover, with the increased concentration of H_2_O_2_, the absorbance of TMB increased greatly, indicating the great amounts of ROS were generated when Si-Pt NCs meet H_2_O_2_. ESR sepetra also proved that Si-Pt NCs with H_2_O_2_ could produce abundant hydroxyl radical **(Figure S5)**. Compared with the group of Si and pure Pt, Si-Pt NCs showed the highest oxidation rate of TMB after adding H_2_O_2_, which reflected that Si-Pt NCs had the stronger peroxidase-like activity than SiNWs and pure Pt NPs **(Figure 2G)**. All the above results revealed that Si-Pt NCs could be used as excellent CDT and SDT agents.

Furthermore, Si-Pt NCs exhibited strong absorption at 1000-1200 nm spectral range **(Figure S6)**, demonstrating the fantastic absorbance of Si-Pt NCs in the NIR-II window. To identify whether Si-Pt NCs could act as effective photothermal agents, the Si-Pt NCs were irradiated with 1064 nm for 5 min with the power density of 1.0 W▪cm^-2^. Si-Pt NCs showed a great concentration-dependent heating effect after 1064 nm laser irradiation** (Figure 2H)**. The temperature of Si-Pt NCs (0.05 mg∙mL^-1^) could increase to 54.2 °C from 23.5 °C maximumly **(Figure S7)**. According to the previous calculation method 45, the photothermal conversion efficiency of Si-Pt NCs was measured to be ~47.7% **(Table S1)**. The fantastic absorbance of Si-Pt NCs in the NIR-II window determined their remarkable photothermal performance. PTT-SDT/CDT combined therapy has been proved to be effective method in many aspects. The mild photothermal effect can increase tumor blood flow, an thereby improving tumor oxygenation to boost the ROS generation from CDT and SDT 46. In addition, the slight temperature increment is able to improve the SDT and CDT catalytic performance and increase the ROS generation 47. From the temperature profiles of Si-Pt NCs with five cycles, no obvious changes were observed during each cycle **(Figure S8)**, which implied that Si-Pt NCs had good photothermal stability. Furthermore, when the temperature increased from 25 ℃ to 45 ℃ under laser irradiation, both the activities of CDT and SDT of Si-Pt NCs were greatly improved **(Figure 2I, 2J, S9)**. To clarify the reason why Si-Pt NCs had such good SDT performance, the solid ultraviolet spectra of Si-Pt NCs and SiNWs were measured **(Figure 2K)**. According to the Tauc plot formula, the band gap of SiNWs was ~1.76 eV, while that of Si-Pt NCs was declined to ~0.55 eV. These results meant that Si-Pt NCs required lower energy for the electrons excited from the valence band (VB) to the conduction band (CB) compared with SiNWs. So it was easier for Si-Pt NCs to enhance the separation efficiency of e^+^- h^-^ under US excitation. With regard to the reason why the chemodynamic effect of Si-Pt NPs was better than that of pure Pt NPs. We proposed the possible mechanism. Both Si-Pt NCs and Pt NPs had good CDT effect, however, due to the mesoporous structure of SiNWs after HF etching, Pt NPs with high catalytic performance could be uniformly arranged on the surface of SiNWs in Si-Pt NCs, avoiding the aggregation of Pt nanoparticles. From the above results, the mild photothermal effect of Si-Pt NCs could enhance the activity of CDT and SDT **(Figure 2L)**.

**Figure 3:**
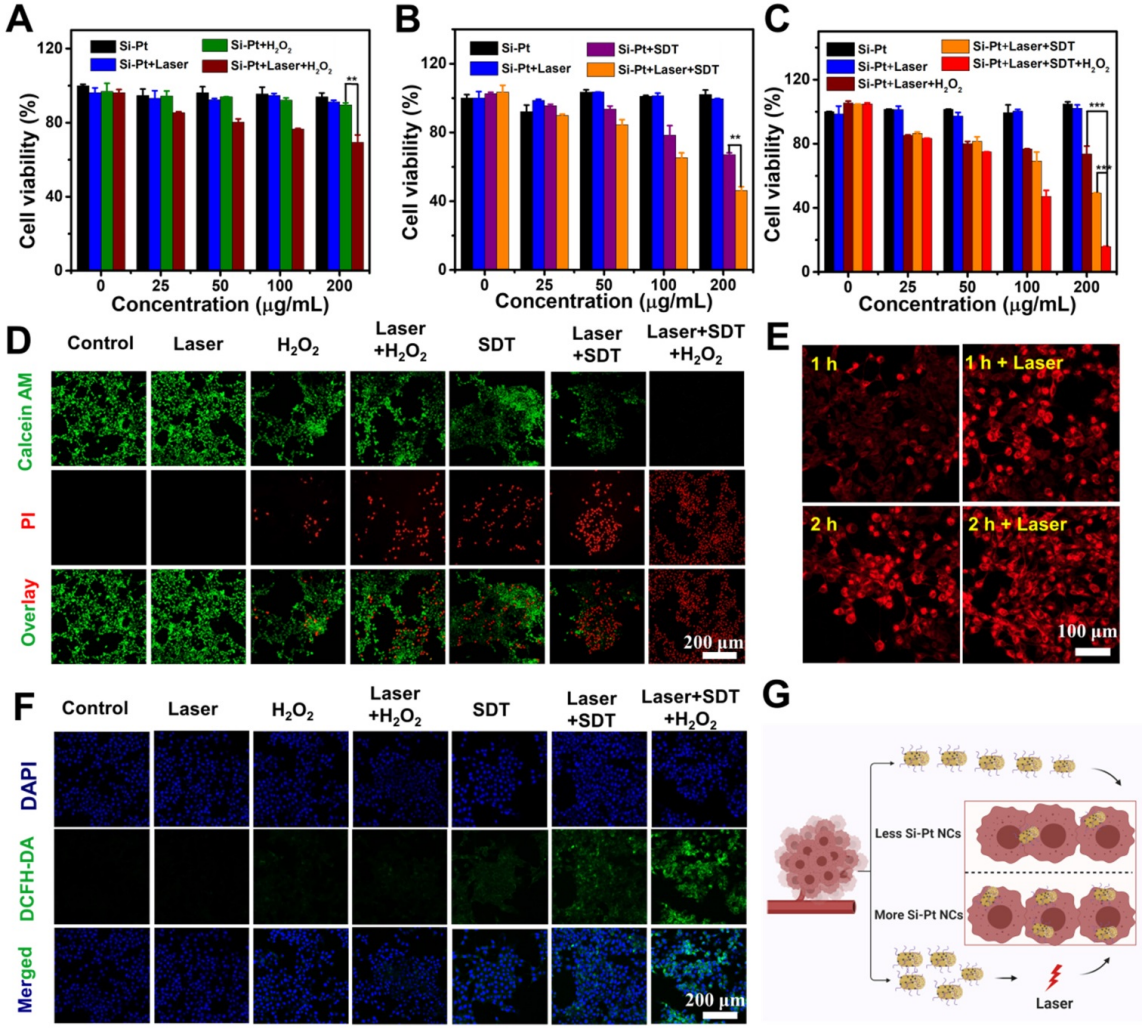
***In vitro* CDT, SDT and PTT of Si-Pt NCs.** Relative viability of 4T1 cells after incubation with different concentrations of **(A)** Si-Pt NCs and 50 μM H_2_O_2_, **(B)** Si-Pt NCs and US irradiation, **(C)** Si-Pt NCs, 50 μM H_2_O_2_ and US irradiation in the presence or absence of 1064 nm laser.** (D)** Confocal images of 4T1 cells stained with Calcein AM (green, live cells) and PI (red, dead cells) after different treatments; **(E)** Confocal images of 4T1 cells incubated with Si-Pt-Cy5.5 NCs after with or without laser irradiation; **(F)** Confocal images of 4T1 cells stained with DCFH-DA after various treatments; and **(G)** A possible schematic diagram showing photothermal enhanced cellular uptake of Si-Pt NCs. P value: *P < 0.05, **P < 0.01, ***P < 0.001.

In order to validate *in vitro* photothermal enhanced SDT and CDT efficiency of Si-Pt NCs, 4T1 mouse breast cancer cells were incubated with different concentrations of Si-Pt NCs and then determined by the standard methyl thiazolyl tetrazolium (MTT) assay. As we can see from **Figure 3A**, Si-Pt NCs had no obvious toxicity to 4T1 cells even at a high concentration (200 μg▪mL^-1^). When the system was added with 25 μM H_2_O_2_, the cell viability of 4T1 cells also had no significant decrease. However, with the 1064 nm laser exposure, the cell viability of 4T1 cells with the concentration of Si-Pt NCs at 200 μg▪mL^-1^ was reduced to ~70%, which had a significant difference from the group of Si-Pt + H_2_O_2_. It could be found that the photothermal effect of Si-Pt NCs could improve the CDT effect. Cells treated with Si-Pt NCs under US irradiation revealed the uniform phenomenon** (Figure 3B),** demonstrating that the efficiency of SDT of Si-Pt NCs could also be improved by the photothermal effect. When ensured that 1064 nm laser had no killing effect on the 4T1 cells, the group of Si-Pt + laser + SDT + H_2_O_2_ showed the lower cell viability than that of the groups of Si-Pt + laser + H_2_O_2_ and Si-Pt + laser + SDT, indicating that the mild photothermal effect of Si-Pt NCs could enhance the efficacy of CDT and SDT of themselves **(Figure 3C)**. Furthermore, the cell-killing effect of Si-Pt NCs with various treatment was determined by live/dead staining (Calcein AM/PI). Si-Pt NCs treated with laser only or H_2_O_2_ only showed almost no hurt to 4T1 cells, but more cells were defunct in the Si-Pt + laser + H_2_O_2_ group. The cells in Si-Pt + US group were partly killed, and more cells were damaged with the 1064 nm laser irradiation. Notably, the group Si-Pt + laser + SDT + H_2_O_2_ showed the strongest red fluorescence from PI among all groups **(Figure 3D)**. As shown in **Figure S10**, the 4T1 cell viability of this group declined to less than 10%. These results illustrated that Si-Pt NCs had significant sono-toxicity against 4T1 cells and the cell lethality resulted from CDT and SDT of Si-Pt NCs increased in the presence of the photothermal effect.

In order to indentify the intracellular process of Si-Pt NCs acting as CDT and SDT agents, 2,7-diclorofluorescein diacetate (DCFH-DA) staining assay was conducted to detect ROS levels in cells** (Figure 3F)**. The tumor cells in the groups of Control, Si-Pt + laser, Si-Pt + H_2_O_2_, and Si-Pt + laser + H_2_O_2_ showed week green influence, which represented the presence of ROS. However, cells treated with Si-Pt + SDT, Si-Pt + laser + SDT, and Si-Pt + laser + H_2_O_2_ + SDT showed stronger green fluorescence owing to the generation of abundant ROS, and the group of Si-Pt + laser + H_2_O_2_ + SDT had the most ROS generation, indicated that Si-Pt NCs as efficient CDT and SDT sensitizers were capable of generating more ROS to kill cells under laser irradiation. In order to monitor the track of Si-Pt NCs in cells, Si-Pt NCs were marked with Cy5.5 dyes, and the amounts of them entering the cells with and without laser irradiation were observed by confocal fluorescence imaging and flow cytometry **(Figure 3E, S11)**. As shown in **Figure 3G**, an increasing level of Si-Pt NCs could enter the cells after 1064 nm laser irradiation, which then increased the efficiency of CDT and SDT treatment at the cellular level. All these results demonstrated that Si-Pt NCs had an excellent photothermal performance enhanced CDT and SDT combined therapy.

**Figure 4:**
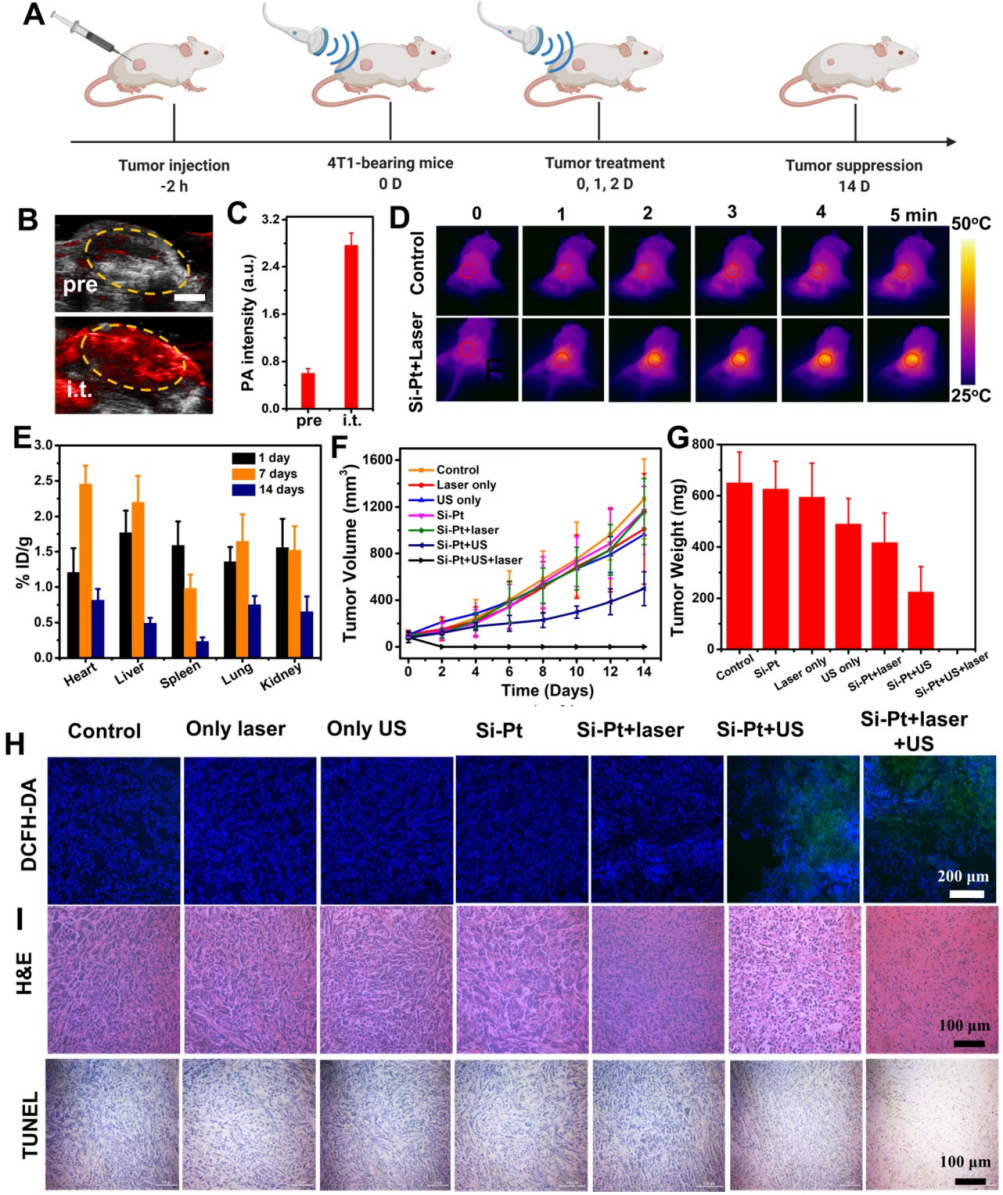
***In vivo* combined therapy**. **(A)** Schematic of the *in vivo* combined therapy on mice. (**B**) PA imaging of tumors in transverse section at 808 nm after intratumoral injection of Si-Pt NCs at the concentration of 1 mg▪mL^-1^. Scale bar: 3 mm. **(C)** PA intensity of tumors intratumorally injected with Si-Pt NCs under the irradiation of a pulse laser of 808 nm. **(D)** IR images after intratumoral injection of Si-Pt NCs under the laser irradiation (1.0 W▪cm^-2^, 5 min); **(E)** Biodistribution of Si-Pt NCs in mice at different days post-intratumor injection; 4T1 tumor **(F)** growth curve and **(G)** tumor weight; **(H)** Fluorescence images of DCFH-DA stained tumor slices collected from mice 24 h post treatment; and **(I)** Microscopy images of H&E and TUNEL stained tumor slices.

Intravenous injection is the most common method of administration of nano-biomaterials drugs for tumor treatment. However, due to the large size and poor blood circulation, the tumor accumulation effect of Si-Pt NCs was not obvious. Therefore, we chose intratumoral injection to prove the concept of the SDT&CDT concept based on Si nanowires. To achieve more accurate SDT and CDT of cancer therapy, it was helpful to apply imaging guidance to allow ultrasound irradiation focusing on the tumor and reducing possible damage to the normal tissues. Since the synthesized Si-Pt NCs had good optical absorbance in the second near-infrared window, they were able to apply for photoacoustic (PA) imaging. 4T1 tumor-bearing mice were intratumorally injected with Si-Pt NCs (200 μL, 5 mg/kg), and the PA image was acquired from a photoacoustic computed tomography scanner at 808 nm. The PA intensity of the tumor after injection was much stronger than that of the pre-injection tumor **(Figure 4B).** Compared with the untreated group, the PA signal increased about 4 folds **(Figure 4C)**, clearly demonstrating the ability of PA imaging of Si-Pt NCs. Encouraged by the photothermal enhanced SDT and CDT efficiency of Si-Pt NCs, we then investigated the effects of treatment *in vivo* of Si-Pt NCs. Mice bearing 4T1 tumors were divided into 7 groups: (1) PBS; (2) Si-Pt only (i.t. injection 15 mg/kg); (3) Laser only; (4) US only; (5) Si-Pt + US; (6) Si-Pt + Laser; (7) Si-Pt + Laser + US. At 2 h after i.t. injection, the tumors were treated with US irradiation or laser + US irradiation, which was repeated once a day in the first three days **(Figure 4A)**. Under the laser irradiation, the temperatures of the tumors were recorded. The temperature could reach to about 50 ℃ within 5 min, while that of the control group changed inconspicuously **(Figure 4D)**. Then we studied the biodistribution of Si-Pt NCs at different times after i.t. injection based on the Si element measured by ICP-OES **(Figure 4E)**. At one day after injection, Si-Pt NCs were hardly retained in the main organs of mice and Si levels in these organs declined quickly over time, showing that Si-Pt NCs had little damage to the main organs. After the treatment, tumor volume and weight were measured every other day. The tumor growth of Si-Pt + US groups was remarkably suppressed after 14 days' therapeutic period, and the tumors treated with the combined therapy even disappeared **(Figure 4F-G, Figure S12),** while the tumors in other groups had a wild growth, which demonstrated that the mild photothermal effect enhancing CDT and SDT had a good therapeutic effect on tumors. Then the liver, spleen, kidney, heart, and lung were collected for hematoxylin and eosin (H&E) staining **(Figure S13)**, there was no significant damage to the mice, indicating that the Si-Pt NCs had good biocompatibility.

*In vivo* ROS staining was conducted to evaluate the ROS production inside the tumors from different groups. In comparison to the other five groups, the ROS signal of Si-Pt + US group and Si-Pt + laser + US group appeared higher, showing stronger green fluorescence. Moreover, the signal seemed to be much stronger under the the US and laser irradiation, suggesting that the US process of Si-Pt NCs could generate high-level ROS, and the laser irradiation could enhance the level of ROS generation **(Figure 4H)**. The photothermal enhanced combined therapy efficacy was also assessed by H&E and TdT-mediated dUTP Nick-End (TUNEL) staining of the tumors after the various treatments **(Figure 4I)**. The tumors from Si-Pt + laser + US group had the most fearful damages compared with other groups, which was consistent with the above tumor growth. All these results demonstrated that Si-Pt NCs could be applied for SDT and CDT, and the mild photothermal effect could enhance the ROS generation for better-combined therapy.

## Conclusions

In summary, Si-Pt NCs were obtained by *in situ* reduction of Pt NPs on SiNWs. Under the treatment of ultrasound, they could produce abundant ROS, which had the effect of SDT. And Si-Pt NCs had good chemodynamic therapy activity, which could convert excess hydrogen peroxide into ROS in the tumor microenvironment. Since the uniform size and distribution of Pt NPs loaded on SiNWs, the SDT and CDT effects of Si-Pt NCs were stronger than these of pure Pt NPs and SiNWs. Both *in vivo* and *in vitro* experiments proved that Si-Pt NCs could be applied for combined SDT/CDT, which had a significant inhibitory effect. In addition, the gentle photothermal effect of Si-Pt NCs could significantly improve the combined treatment effect of SDT/CDT. These developed Si-Pt NCs would provide novel platforms for combined cancer therapy.

## Supplementary Material

Supplementary materials and methods, figures, and table.
