# Supplementary material for: Silicon nanowires decorated with platinum nanoparticles were applied for photothermal-enhanced sonodynamic therapy
Source: Theranostics. 2021 Sep 3;11(19):9234–42. doi: 10.7150/thno.58755 (PMC8490511; doi:10.7150/thno.58755)
Supplement: Supplementary file 1 — Supplementary materials and methods, figures, and table. [file thnov11p9234s1.pdf]

# Supporting Information

## Silicon nanowires decorated with platinum nanoparticles were applied for photothermal-enhanced sonodynamic therapy

Lina Sun, Xianwen Wang, Fei Gong, Kui Yin, Wenxiang Zhu, Nailin Yang, Shang Bai, Fan Liao, Mingwang Shao*, and Liang Cheng*

Institute of Functional Nano & Soft Materials Laboratory (FUNSOM), Collaborative Innovation Center of Suzhou Nano Science and Technology, Soochow University, Suzhou, Jiangsu 215123, China

E-mail: lcheng2@suda.edu.cn; mwshao@suda.edu.cn

# Materials and methods

## Materials

Fetal bovine serum (FBS) and Roswell Park Memorial Institute 1640 medium (RPMI-1640) were purchased from Gibco BRL (Eggenstein, Germany). $HPtCl_6·6H_2O$ was purchased from Aladdin Industrial Co. Hydrogen fluoride (HF, 40%) was obtained from Sinopharm Chemical Reagent Co., Ltd. 3,3,5,5-Tetramethylbenzidine (TMB), 1, 3-diphenylisobenzofuran (DPBF) o-Phenylenediamine (OPD) and 2,7-diclorofluorescein diacetate (DCFH-DA) were obtained from Sigma-Aldrich. All other chemical reagents were analytical grade and were used without further purification.

## Preparation of SiNWs

SiNWs were prepared by a thermal-evaporation oxide-assisted growth method *via* the established protocols. In brief, SiO powder (Aldrich, 325 mesh, 99.9%) on an alumina boat was placed at the center of a horizontal alumina tube mounted inside a high-temperature tube furnace. The system was evacuated to a pressure of $1×10^2$ Torr. A carrier gas consisting of 95% Ar and 5% $H_2$ was introduced and maintained at a pressure of 10 Torr with a constant flow rate of 150 standard-state cubic centimeter per minute (sccm). The furnace was heated to 1300 °C at the heating rate of 40 $°C·min^{-1}$ and maintained at this temperature for 4 h. The products were collected on the inner surface of the alumina tube.

## Synthesis of Si-Pt

SiNWs (30 mg) and 2 mL of pure water were put in a mortar to grind and stirred using a magnetic stirrer for 5 min to get a well-distributed suspension. Hydrofluoric acid (HF) solution (800 μL, 5%) was added to the suspension, which was stirred for 10 min to remove the thin oxide layer and form silicon–hydrogen (Si–H) bonds on the SiNWs. Afterwards, 3 mL of 10 mg/mL $HPtCl_6·6H_2O$ was added. The mixture was stirred for 10 min at room temperature and rinsed with ultra-pure water three times. In

this way, Pt NPs were reduced *via in situ* reduction and grown on the surface of SiNWs. In the control experiment, pure Pt NPs were obtained by etching SiNWs of Si–Pt *via* excessive 40% HF aqueous solution for 2 h at a room temperature.

**Modification of Si-Pt**

For surface modification, Si-Pt was conjugated with Polyethylene Glycol-Thiol (PEG-SH) *via* Pt–S bonds. Si-Pt (1.0 mg) was dispersed in 5 mL of ultra-pure water and ultra-sonicated for 5 min to give a clear dispersion. And then, 20 mg of PEG-SH was added to it and stirred overnight. Residual PEG-SH molecules were removed by centrifugation at 8000 rpm and washing with ultra-pure water three times.

For marked with Cy5.5 dyes, Si-Pt (1.0 mg) was dispersed in 5 mL of ultra-pure water and ultra-sonicated for 5 min to give a clear dispersion. And then, 20 mg of SH-PEG-$NH_2$ was added to it and stirred overnight. Residual SH-PEG-$NH_2$ molecules were removed by centrifugation at 8000 rpm and washing with ultra-pure water three times. Then, amino activation Cy5.5 dyes (10 μL) were add to Si-Pt-PEG-$NH_2$ solution and stirred overnight. Residual Cy5.5 dyes were removed by centrifugation at 8000 rpm and washing with ultra-pure water three times.

**Materials characterization**

The phase and crystallographic structure of all as-prepared samples were characterized by X-ray powder diffraction (XRD, Philips X'pert PRO MPD diffractometer) equipped with a $CuK_a$ radiation (= 0.15406 nm) source. Transmission electron microscopy (TEM) and high-resolution TEM (HRTEM) were conducted on a FEI Tecnai F20 transmission electron microscope with an accelerating voltage of 200 kV. Energy-dispersive X-ray spectroscopy (EDX) mapping was obtained using scanning electron microscopy (SEM) (Zeiss Supra 55). X-ray photoelectron spectroscopy (XPS) was undertaken using a Kratos AXIS UltraDLD ultrahigh vacuum surface analysis system with Al $K_a$ radiation (1486 eV) as a probe and an indium plate as the sample holder. UV-vis-NIR spectra were recorded on a

PerkinElmer Lambda 950 UV-vis-NIR spectrophotometer.

**FTIR monitoring the Si-H bonds formation**

For control, 3 mg silicon nanowires were added into 50 mg KBr and grinded for some minutes, then it was made into sample of FTIR. For Si-H bonds testing, 3 mg silicon nanowires and 5 μL 5% HF solution were added into 50 mg KBr and grinded for some minutes and then made into sample [1].

**Quantitative analysis of the generation of $^1O_2$ and •OH in SDT**

For $^1O_2$ detection in SDT, 1 mL Si-Pt (40 μg/mL based on Pt) were mixed with 20 μL DPBF (1 mg/mL). After different US irradiation (40 kHz, 3 W/cm$^2$) durations, the absorbance changes of DPBF at 420 nm were recorded to quantify the generation rate of $^1O_2$ by Si-Pt. For comparison, pure Pt NPs and pure SiNWs were measured under the same conditions.

For •OH detection in SDT, 10 μL OPD (10.8 mg/mL) was added into 1 mL Si-Pt (40 μg/mL based on Pt). After different US irradiation (40 kHz, 3 W/cm$^2$) durations, the absorbance changes of OPD at 420 nm were monitored to quantify the generation rate of •OH by Si-Pt. For comparison, pure Pt NPs and pure SiNWs were measured under the same conditions.

***In vitro* detection of ROS from CDT**

For the detection of •OH generation from CDT, Si-Pt dispersed in $H_2O_2$ aqueous solutions (0, 25, 50, 100 μM) were mixed with 4 μL TMB (80 mM). The absorbance changes of TMB at 654 nm were monitored to quantify the generation rate of •OH from CDT. For comparison, pure Pt NPs and pure SiNWs were measured under the same conditions.

Because the activity of TMB and OPD would not be affected by temperature, they were used as indicators to monitor the influence of different temperatures on CDT and SDT activity, respectively [2]. In detail, for CDT, Si-Pt NCs, TMB and $H_2O_2$

(50 μM) were incubated for 30 min at room temperature (25 °C). UV-vis-NIR spectra were measured. The catalytic performance of Si-Pt NCs at 45 °C was further explored to simulate the effect of catalytic property by the mild photothermal treatment. For SDT, Si-Pt NCs and OPD were incubated for 30 min at room temperature (25 °C) and then were irradiated by US for 2 min. UV-vis-NIR spectra were measured. The SDT performance of Si-Pt NCs at 45 °C was further explored to simulate the effect of SDT by the mild photothermal treatment.

**Cellular experiment**

Murine breast cancer 4T1 cell were purchased from American Type Culture Collection (ATCC) and cultured in the standard cell culture medium at 37 °C under 5% $CO_2$. For *in vitro* SDT, 4T1 cells seeded in 96-well plates were incubated with Si-Pt at various concentrations for 8 h, followed by US irradiation (40 kHz, 3 W/cm$^2$, 2 min). For photothermal enhanced CDT-SDT experiment, 4T1 cells seeded in 96-well plates were incubated with Si-Pt at various concentrations for 4 h and then irradiated with 1064 nm laser (1 W/cm$^2$, 10 min). After that, cells were incubated for another 4 h. Then nanomaterials were removed and $H_2O_2$ aqueous solutions (50 μM) were added, followed by US irradiation (40 kHz, 3 W/cm$^2$, 2 min). The cell viabilities were determined by a standard methyl thiazolyl tetrazolium (MTT) assay.

For fluorescence imaging, 4T1 cells after various treatments were further stained by Calcein AM/propidium iodide (PI, Sigma-Aldrich). For the detection of intracellular ROS, cells were incubated with 2, 7-dichloro-dihydro-fluorescien diacetate (DCFH-DA, 20 μM, Sigma) for 30 min for confocal fluorescence imaging (CLSM, Zeiss Axio-Imager LSM-800).

**Tumor model**

Female Balb/c mice (4–6 weeks old) were purchased from Suzhou Elmet Technology Co. Ltd (Suzhou, China) and used under the protocols approved by Soochow University Laboratory Animal Center. $2\times10^6$ 4T1 cells in 50 μL (PBS) were

subcutaneously injected into the back of each female Balb/c mouse.

**PA imaging *in vivo***

For *in vivo* PA imaging, mice were anesthetized and injected with Si-Pt intratumorally. 2 h later, the images of PA imaging in vivo were obtained from a photoacoustic computed tomography scanner (Endra's Nexus 128, Ann Arbor, MI).

***In vivo* cancer therapy**

Mice bearing 4T1 tumors (~100 $mm^3$) were divided into seven groups (n=5 per group): (1) PBS; (2) Si-Pt only (i.t. injection 15 mg/kg); (3) Laser only (1064 nm, 1 $W/cm^2$, 5 min); (4) US only (40 kHz, 3 $W/cm^2$, 5 min, 50% duty cycle); (5) Si-Pt + US; (6) Si-Pt + Laser; (7) Si-Pt + Laser + US. Si-Pt NCs were intratumorally injected into mice bearing 4T1 tumors. At 2 h after i.t. injection, the tumors were treated with US irradiation, which was repeated every day for three times. Tumor sizes and body weights were monitored every two days. The tumor volumes were calculated by the formula: volume = $length \times width^2/2$. For H&E staining and TUNEL staining, tumors with different treatments on the second day were collected. Meanwhile, ROS from tumors was also detected by DCFH-DA.

## Supporting Figures

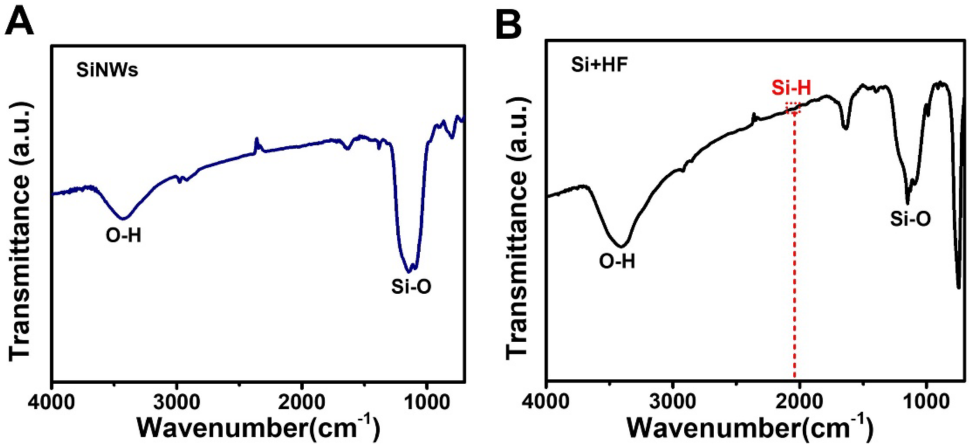


**Figure S1.** FTIR of (**A**) SiNWs and (**B)** SiNWs treated with 5% HF solution. The peak ranging from 2300 to 1900 $cm^{-1}$ was contributed to Si-H bonds.

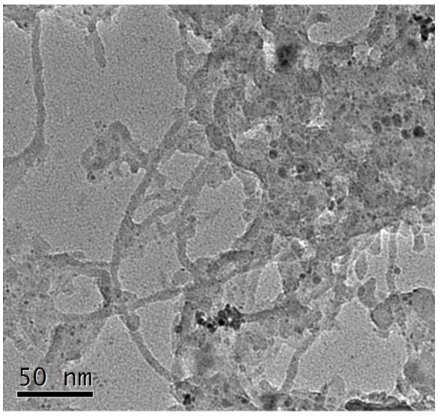


**Figure S2.** TEM image with large magnification of Si-Pt NCs.

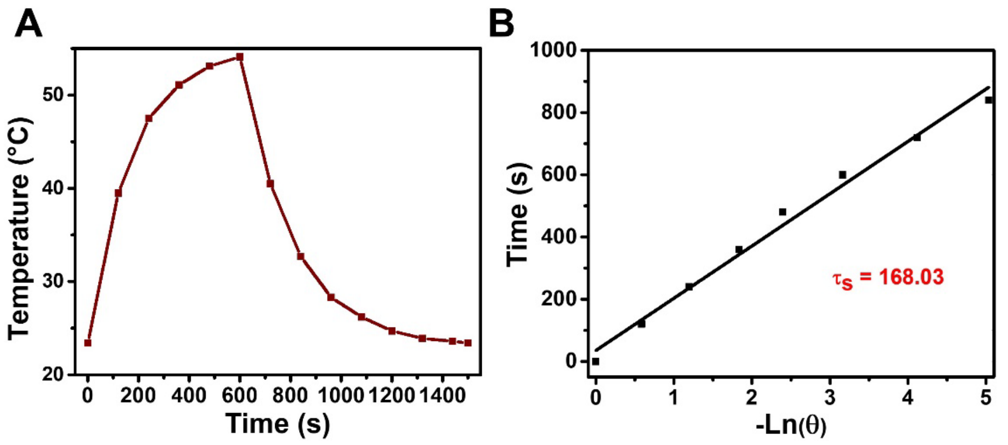


**Figure S3.** Hydrodynamic diameters of Si-Pt NCs in different solutions including DI water, PBS, normal saline, and RPMI 1640 cell culture medium (containing 10% serum) after (**A**) one day and (**B**) seven days.

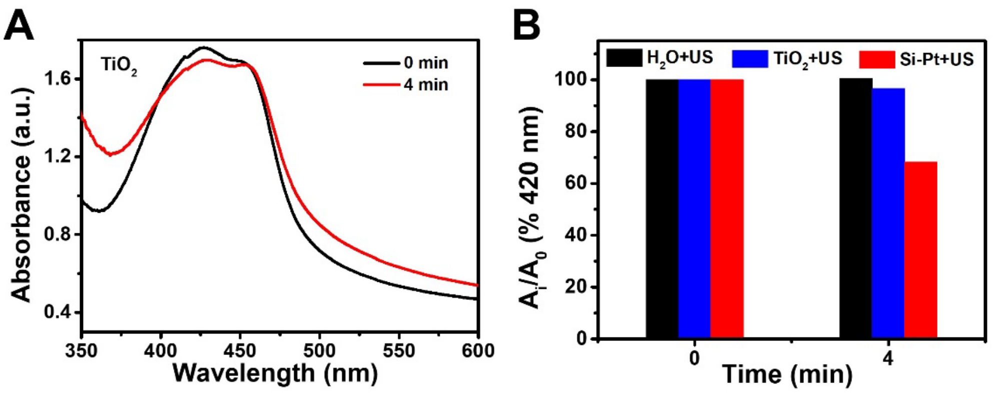


**Figure S4.** (**A**) DPBF oxidation by $TiO_2$ nanoparticles under US irradiation; and (**B**) Comparison of DPBF oxidation by Si-Pt and $TiO_2$ under US irradiation for 4 min.

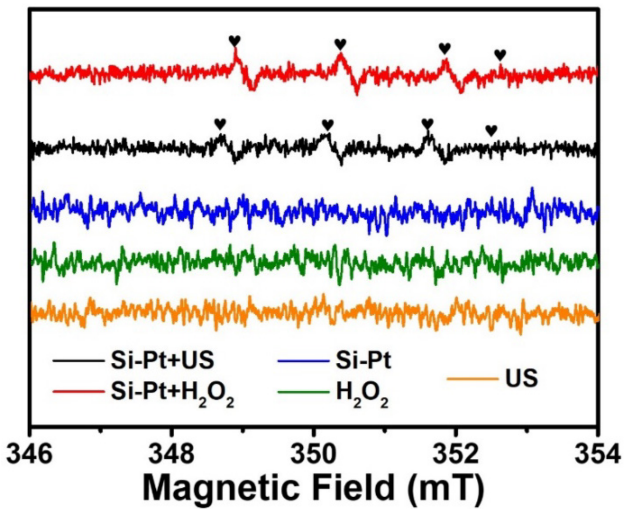


**Figure S5.** ESR spectra of DMPO/•OH adducts under different conditions.

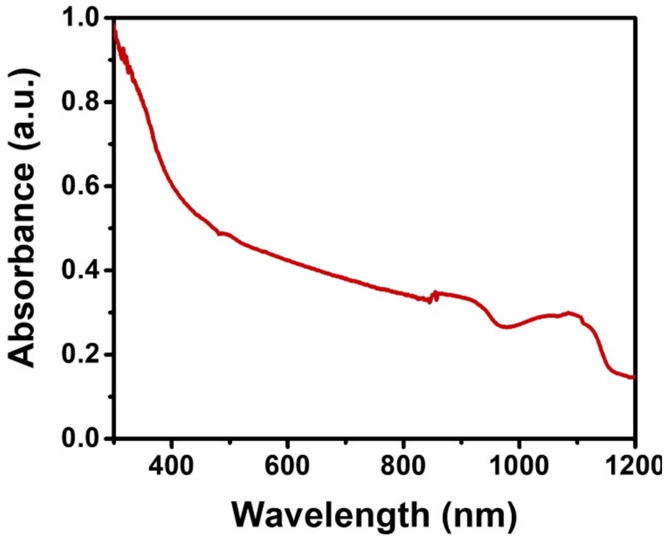


**Figure S6.** UV-vis-NIR absorption spectra of Si-Pt NCs.

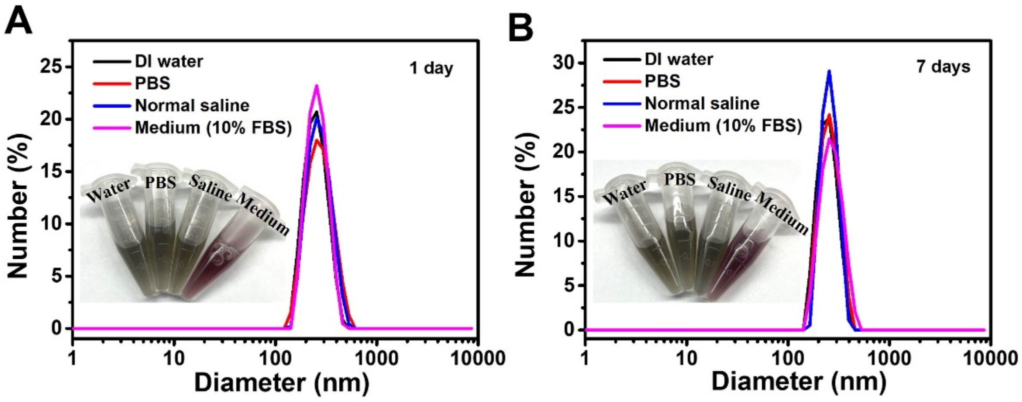


**Figure S7.** (**A**) Temperature profile of Si-Pt NCs (0.05 mg·mL$^{-1}$) irradiated by a 1064 nm laser (1.0 W·cm$^{-2}$) for 10 min followed by natural cooling when the laser is turned off; (**B**) measuring the time constant for heat transfer from the system using a linear regression of the cooling profile.

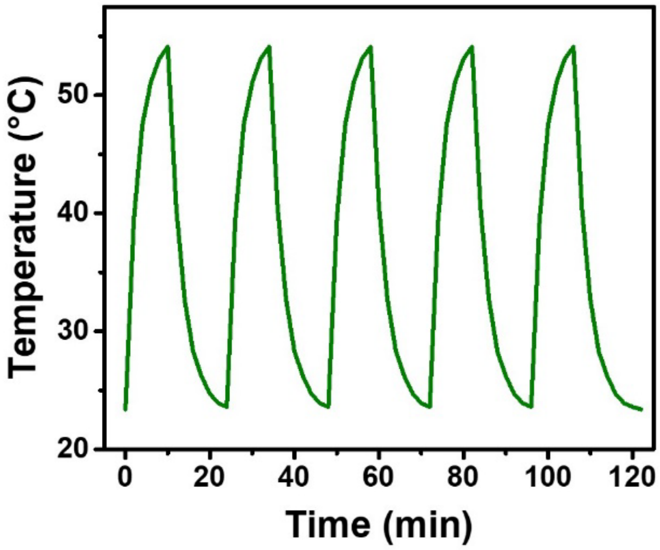


**Figure S8.** Repeated heating/cooling profiles of Si-Pt under the irradiation of 1064 nm laser on/off (1.0 W·cm$^{-2}$).

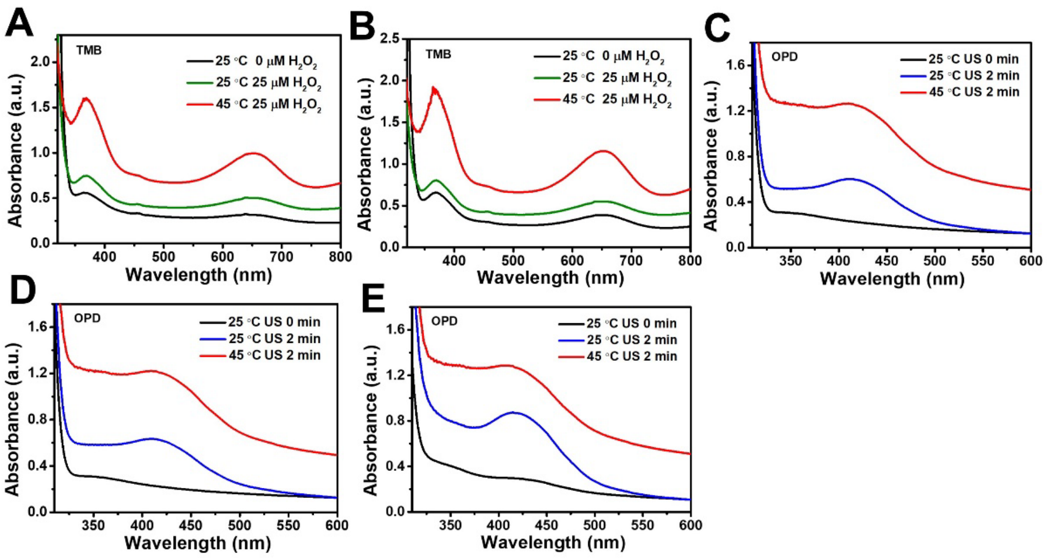


**Figure S9.** (**A-C**) The oxidation of TMB by Si-Pt NCs with the addition of $H_2O_2$ and (**D**, **E**) the OPD oxidation under US irradiation for 2 min at different temperature (25 °C; 45 °C) under the control of laser irradiation.

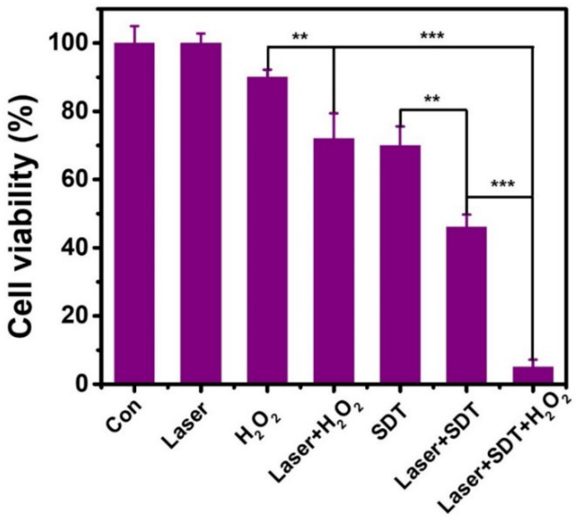


**Figure S10.** Relative cell viability of 4T1 cells after different treatments in Figure 3D. P value: *P < 0.05, **P < 0.01, ***P < 0.001.

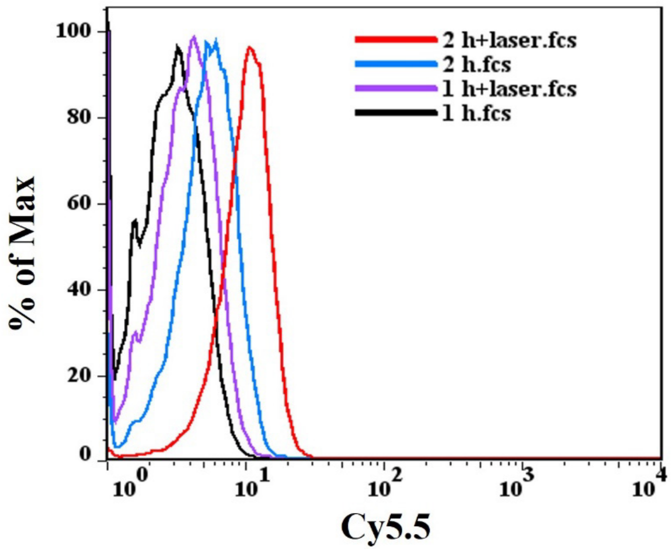


**Figure S11.** Quantitative analysis of cellular uptake by Flow Cytometry in Fig 3E.

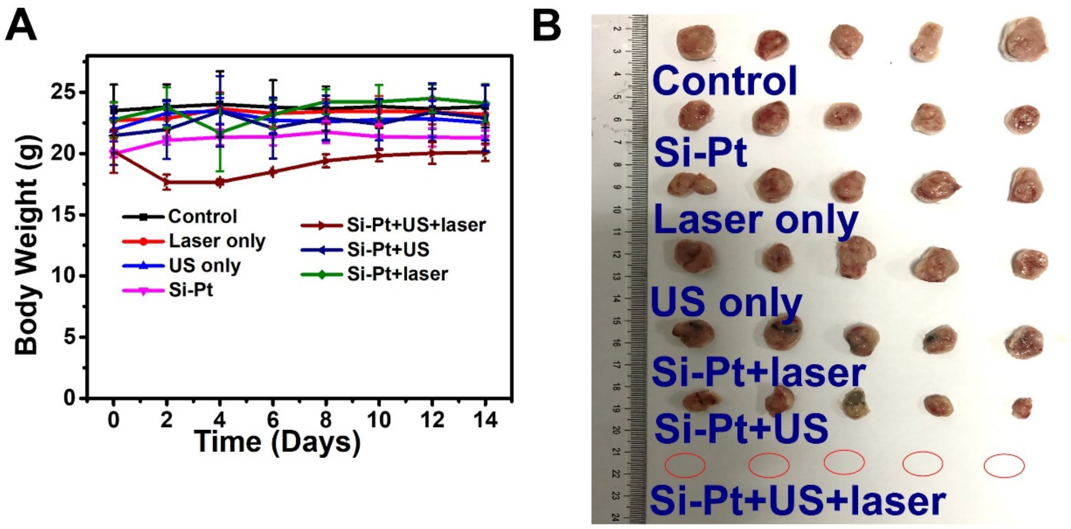


**Figure S12.** (**A**) Mice body weights and (**B**) tumor images after various treatments.

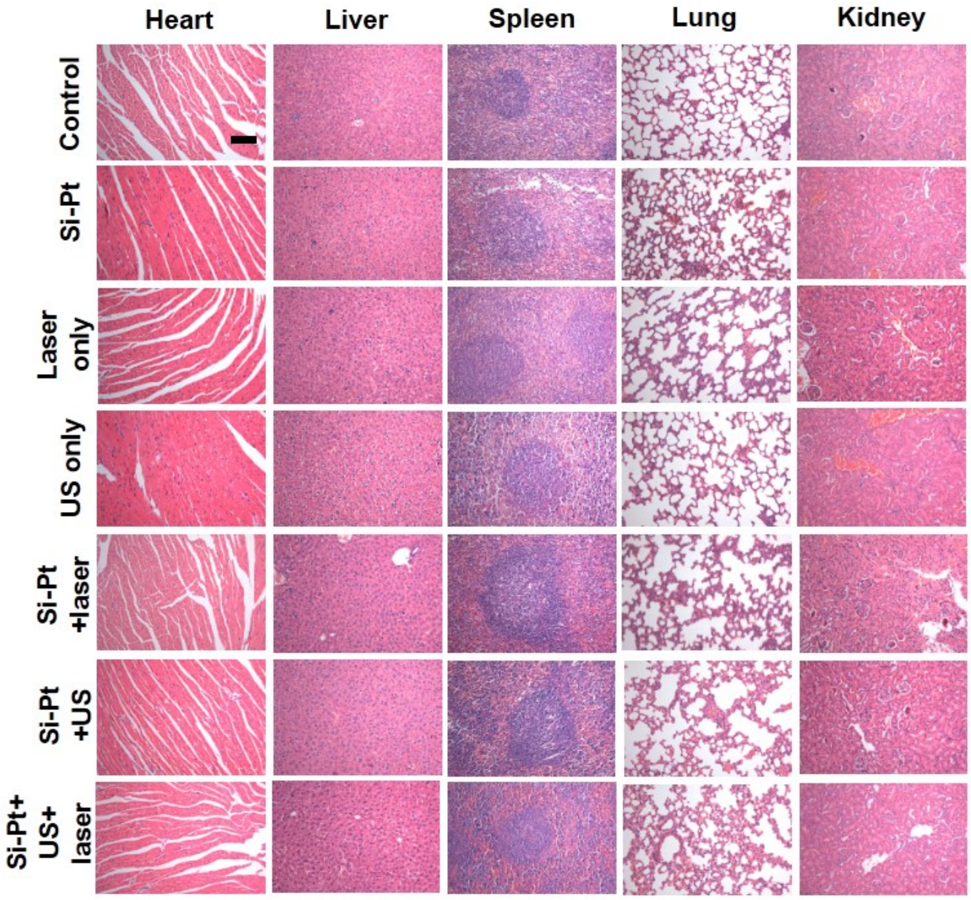


**Figure S13.** Microscopy images of H&E-stained different organs (including heart, liver, spleen, lung, and kidneys) of the different groups 14 days post-treatment (Scale bare: 200 μm).

**Table S1.** Parameters for photothermal conversion efficiency calculation.

| Parameters | Si-Pt NCs |
|---|---|
| m (g) | 0.5 |
| C ($J{\cdot}g^{-1}$) | 4.2 |
| $\tau_s$ (s) | 168.03 |
| $T_{max}$ (system) (°C) | 54.2 |
| $T_{max}$ ($H_2O$) (°C) | 30.4 |
| I (W) | 1.0 |
| $A_{1064}$ | 0.29 |
| ŋ | 0.477 |

## References

1. Fan Z, Liao F, Shi H, Liu Y, Dang Q, Shao M, et al. One-step direct fixation of atmospheric $CO_2$ by Si-H surface in solution. iScience. 2020; 23: 100806.

2. Wang X, Fan L, Cheng L, Sun Y, Wang X, Zhong X, et al. Biodegradable nickel disulfide nanozymes with GSH-depleting function for high-efficiency photothermal-catalytic antibacterial therapy. iScience. 2020; 23: 101281.
